# First intravenous thrombolysis for pCys194Arg Notch 3 mutation in a Moroccan CADASIL patient with stroke

**DOI:** 10.1016/j.radcr.2024.03.006

**Published:** 2024-04-04

**Authors:** Mohamed Amine Mnaili

**Affiliations:** a Neurology Department, Agadir Military Hospital, Agadir, Morocco; b University of Hassan II, Casablanca, Morocco

**Keywords:** CADASIL, Acute stroke, Thrombolysis intravenous tenecteplase

## Abstract

Cerebral autosomal dominant arteriopathy with subcortical infarcts and leucoencephalopathy (CADASIL) is caused by mutations in the NOTCH3 gene. Clinical manifestations of CADASIL include lacunar infarcts, transient ischemic attacks, dementia, migraine, and psychiatric disorders.

Cerebral MRI can show signal abnormalities in the basal ganglia and white matter, especially characteristic when located in the anterior part of the temporal lobe and external capsules. We report CADASIL patient treated with intravenous tenectelase for acute ischemic stroke, and we present a review of literature aimed to report effectiveness and safety of intravenous thrombolysis in CADASIL patients.

## Introduction

CADASIL is a hereditary small vessel disease caused by mutations in the NOTCH3 gene, leading to toxic NOTCH3 protein accumulation in the small- to medium sized arterioles. It is the most common monogenic cause of stroke and is clinically characterized by migraine with aura, stroke, psychiatric disturbances, acute reversible encephalopathy, and cognitive impairment [1]. On magnetic resonance imaging (MRI), CADASIL is characterized by diffuse white matter T2 hyperintensities, multiple lacunes, and microbleeds [1].

We report a case of a patient presenting with stroke and extensive white matter disease on brain MRI, with a history of diabetes mellitus that was shown to be due to CADASIL treated with intravenous thrombolysis.

## Case présentation

A 48-year-old man with a medical history including diabetes mellitus for which he was on metformin who had complained for 3 years of recurrent episodes of migraine attacks, most often accompanied by a flickering scotoma-like aura and cheiro-oral paresthesias. The patient had no signs of intracranial hypertension or extra-neurological signs.

The patient also describes a history of progressive weakness of the right lower limb for 6 months. His father had been suffered by ischemic strokes; his nephew also had CADASIL and had symptoms of epilepsy, recurrent stroke, and suffered from migraine. He was admitted to the emergency room due to sudden worsening of right hemibody weakness and loss of speech. On admission, the neurological examination showed mild right hemiparesis and motor aphasia. The National Institute Health Stroke Scale (NIHSS) score was 7.

**Fig. 1:**
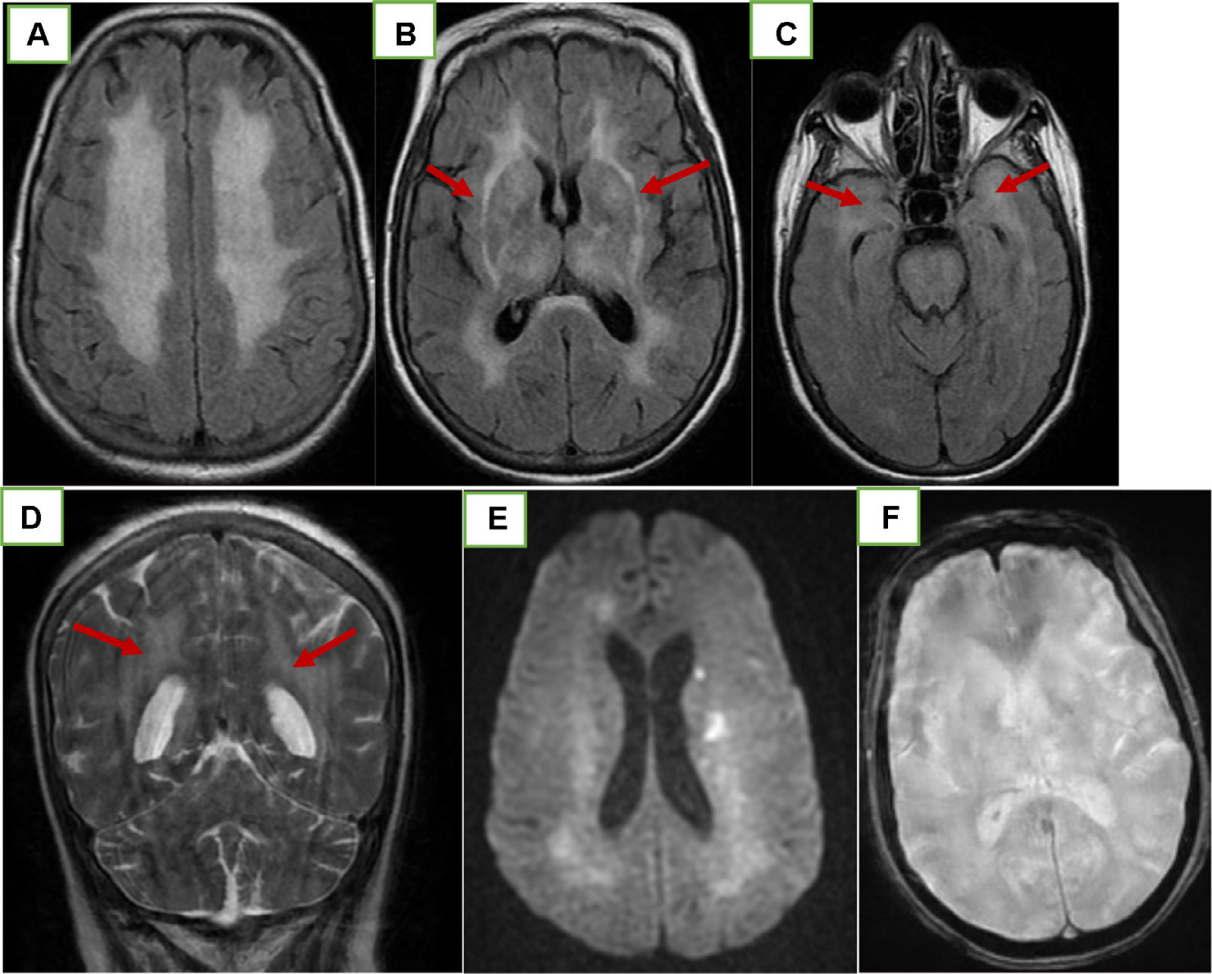
Cranial MRI on axial FLAIR sequence (A to C) and coronal T2 weighted sequence (D) showing increased signal intensity interesting both anterior temporal lobes, the periventricular regions, the subcortical areas, and the external capsules (arrow). DWI image (E) showing a restricted lesion in the left corona-radiata. T2-weighted images (F) did not show microbleeds.

Brain MRI show acute lesions in the territory of the left middle cerebral artery, with no evidence of bleedings. FLAIR sequences demonstrated multiple hyperintense lesions in the subcortical white matter, basal ganglia, thalami, pons, external capsules, and temporal poles (Fig. 1).

In the absence of contraindications, 145 minutes after the onset of symptoms, intravenous thrombolysis with tenecteplase 0.4 mg/kg was administered as a bolus. The neurological examination clearly improved 10 minutes after thrombolysis to a NIHSS of 2 (slight right hemipareisis).

To exclude inflammatory-immunologic diseases, such as vasculitis, all coagulation tests cited were normal: protein S, protein C, total homocysteine, anti-DNA antibodies, anti-cardiolipin antibodies and lupus anticoagulant. Cerebrospinal fluid analysis and infectious disease screening, did not reveal any pathological finding. Ultrasonography of his extracranial and intracranial arteries was normal.

Taking also into account the family history, CADASIL scale was calculated [2] obtaining a score of 15, and the disease was suspected. We decided to test for NOTCH3 mutations. Sequencing showed heterozygous mutation of exon 4 with C580T>C nucleotide's change and pVys194Arg amino-acids change. Diagnosis of CADASIL was made based on clinical signs, brain MRI findings and genetic testing.

## Discussion

CADASIL is the most common genetic cause of ischaemic stroke, first reported for the first time by Van Bogaert as ‘hereditary Binswanger's disease’ in 1955 [3]. A study from the UK estimates that the prevalence of the disease is 2 per 100 000, but this may be an underestimate, as the disorder is thought to be misdiagnosed [4]. CADASIL patients carry the NOTCH3 gene mutation with an autosomal dominant Mendelian mode of inheritance with variable penetrance. It is a nonamyloid and nonatherosclerotic microangiopathy leading to progressive degeneration of the smooth muscle cells of blood vessels due to the abnormal accumulation of NOTCH3 at the level of the cytoplasmic membrane of these cells [5].

The NOTCH3 protein is a unique transmembrane receptor involved in stem cell differentiation. It is not yet clear how NOTCH3 mutations lead to the pathological changes observed in CADASIL [6].

Clinical features of CADASIL include migraine with aura, ischemic stroke-like events, cognitive decline, and mood changes. Our patient had migraine with aura but no behavioral changes. The existence of motor symptoms in the form of progressive weakness may be attributed to multiple stroke-like ischemic events that the patient may have had in the past. Additional tests to help diagnose CADASIL include genetic testing and skin biopsies.

On brain MRI, FLAIR revealed areas of hyperintensity in bilateral subcortical white matter as well as subcortical lacunar lesions in the anterior temporal lobes. These lesions tend to become confluent and affect other regions of the brain with progression of the disease, notably the basal ganglia and external capsules. These signal abnormalities are not specific and can be observed in other diseases [7,8]. However, involvement of the anterior temporal lobes (86%) and external capsules (93%) is specific to suggest the diagnosis of CADASIL [9,10]. Brain MRI of our patient showed the presence of these features, It also subsequently showed the presence of a new left infarct. Cerebral microhemorrhages in 31%-69% of the patients with CADASIL and are indicator that the disease has progressed to the advanced stage [11]. These were absent on the brain MRI of our patient.

Genetic tests look for the presence of the NOTCH3 mutation, which is found in all patients affected by the disease [12]. It is the reference in terms of CADASIL diagnosis.

There are few reported cases of thrombolysis in CADASIL. The risk of hemorrhage is high in CADASIL patients, mainly due to the presence of microbleeds and the occurrence of spontaneous cerebral hemorrhages, particularly in patients suffering from hypertension. Even if its actual frequency is not known, the occurrence of intracerebral hemorrhages remains rare [13]. Concerning the risk of bleeding after thrombolysis in CADASIL, the available information is insufficient to draw conclusions.

Among the seven reported patients (including ours), no bleedings occurred. All patients had brain lesions on MRI but microbleeds were present only in one.

There is no definitive treatment for CADASIL. Antihypertensive drugs, statins, and antiplatelet agents are the main preventive measures of ischemic attacks. Several factors, including hypertension, gender, and intercurrent infections could reduce the life expectancy of patients with CADASIL [12].

## Conclusion

CADASIL is a pathology to consider in any young patient presenting with multiple ischemic strokes or silent cerebral infarctions affecting mainly, but not only, the small and medium-sized cerebral arteries. Our patient did not present any hemorrhagic complications. We present through a literature review the good clinical results in CADASIL patients treated by intravenous thrombolysis for an acute ischemic stroke.

## Patient consent

Written informed consent was obtained from the patient for publication of the details of their medical case and any accompanying images.
